# Mitochondrial Management of ROS in Physiological and Pathological Conditions

**DOI:** 10.3390/antiox14010043

**Published:** 2025-01-02

**Authors:** Paola Venditti, Gaetana Napolitano

**Affiliations:** 1 Department of Biology, University of Naples Federico II, Monte Sant’Angelo Via Cinthia, 80126 Naples, Italy; 2 Department of Sciences and Technology, University of Naples Parthenope, Via Acton 38, 80133 Naples, Italy

Mitochondria are found, with rare exceptions [1,2], in the vast majority of the cells of eukaryotic organisms, where they perform several essential functions ranging from the control of cellular calcium levels to the oxidation of fatty acids to the metabolism of steroids and amino acids, as well as the synthesis of urea and phospholipids [3,4,5,6]. However, their primary function is to produce much of the energy source that is used in the endergonic reactions of cells, namely adenosine triphosphate (ATP), through processes of electron transport, the formation of a proton gradient straddling the inner mitochondrial membrane, and oxidative phosphorylation [6,7]. Mitochondria are also considered to be relevant sources of reactive oxygen species (ROS) [8,9] and are equipped with an important antioxidant defense system [10,11] to defend against them. This system makes them capable of removing a large part of the ROS they produce and contributing to the removal of ROS produced by other cellular sources [12,13]. Due to their ability to act as ROS sinks, mitochondria can act as modulators of cellular ROS levels [10]. This last function is essential, considering ROS can act as second messenger agents by activating redox-sensitive signals in the pathways that are necessary for adequate cell growth and proliferation [14]. Environmental and hormonal factors can modulate mitochondrial ROS production [15,16] and when cellular ROS production is excessive, mitochondria can be damaged, promoting cellular dysfunction [17,18,19].

In this Special Issue, we delve into the potential of mitochondria in managing ROS in physiological and pathological conditions, offering a hopeful outlook for future research and therapeutic strategies.

ROS can be involved in the action mechanisms of some molecules with antitumoral activity.

Bonnante et al. [20] evaluated the antitumor effects of Shikonin (SHK), a naphthoquinone derivative that is the main component of *Lithospermum erythrorhizo*. SHK has various pharmacological activities, including anti-inflammatory, anti-oxidative, anti-virus, anti-bacterial, and anti-cancer effects. The latter effect appears to be linked to the ability of SHK to induce the production of ROS that modulate cancer survival pathways such as PI3K/AKT/mTOR and MAPK signaling. The authors evaluated the effects of SHK against an aggressive malignant T cell lymphoma—adult T cell leukemia/lymphoma (ATLL)—that develops in some elderly carriers of the human T cell leukemia virus (HTVL-1). The authors reported that the SHK-induced apoptosis of ATLL cells is accompanied by the generation of ROS, the loss of mitochondrial membrane potential, and the induction of endoplasmic reticulum (ER) stress. Treatment with an ROS scavenger—N-acetylcysteine (NAC)—prevented the loss of mitochondrial membrane potential and ER stress, as well as preventing the apoptosis of ATLL cells, suggesting that ROS are factors involved in the tumor suppressor action of SHK. SHK also showed the ability to suppress tumor growth in an ATLL-xenografted mouse model without showing significant adverse effects. Therefore, SHK is a promising agent against ATLL.

The role of mitochondrial dysfunction and the resultant increase in ROS production in exacerbating the effects of high glucose levels and diabetes-related complications is a key area of research that warrants attention.

Diabetic retinopathy is a complication that can lead to blindness in diabetic patients. Mitochondrial ROS production can reduce the efficacy of the transplantation of umbilical-cord-blood-derived mesenchymal stem cells (UCB-MSCs), which is an emerging treatment for diabetic retinopathy. Jo et al. [21] found that the pretreatment of the UCB-MSCs with tacrolimus can improve the therapeutic efficacy of transplantation. Indeed, high glucose conditions can increase calcium levels, mitochondrial fission, mitochondrial ROS accumulation, and the induction of apoptosis. Tacrolimus pretreatment prevented high glucose-induced mtROS levels and apoptosis. The authors also showed that tacrolimus action is mediated by the suppression of the high glucose-induced O-GlcNAcylation and activation of the nuclear factor of activated T cells 1 (NFATC1) signaling, which is essential for dynamin-related protein 1 (DRP1)-mediated mitochondrial fission and mitochondrial apoptosis. Therefore, the authors proposed NFATC1 suppression by tacrolimus as a promising therapeutic strategy to improve the efficacy of UCB-MSC transplantation for diabetic retinopathy.

The progression of type 2 diabetes is linked to endothelial dysfunction, which plays a pivotal role in the onset of diabetes-associated cardiovascular complications.

Martino et al. [22] evaluated the effects of whey, a dairy by-product, on human endothelial cells (TeloHAEC) kept in in vitro diabetic conditions (palmitic acid plus high glucose). Whey contains bioactive compounds (betaines and acylcarnitines) that can modulate the metabolism of the cells by acting on mitochondrial energy metabolism. The authors found that whey protected endothelial cells from cytotoxicity and prevented cell cycle arrest, apoptotic cell death, redox imbalance, and metabolic alterations. Moreover, whey treatment counteracted mitochondrial injury and restored sirtuin 3 (SIRT3), which, in endothelial cells, inactivates ROS, subsequently emerging as a relevant contributor to endothelial energy homeostasis. Indeed, the authors found that by treating cells with siRNA, which mediates the suppression of SIRT3, the protective effects of whey on the mitochondrial and metabolic impairment caused by diabetic conditions were abolished. These studies underlined the role of whey as a redox and metabolic modulator in the diabetic state.

ROS and mitochondrial dysfunctions have been associated with several brain injuries.

Alterations in nervous cell functions can originate from traumatic brain injury (TBI), which can last for days, weeks, or months. Brain injury provokes several harmful effects, including the increased release of excitatory neurotransmitters (glutamate, aspartate), alteration in glycemic homeostasis, mitochondrial dysfunction, and oxidative stress onset. Lazzarino et al. [23] evaluated the effects of brain CoQ levels and its redox state in male rats subjected to mild and severe traumatic brain injury (mTBI and sTBI, respectively). Seven days after the damage, the authors assessed the CoQ9, CoQ10, and α-tocopherol levels. The levels of CoQ9 and α-tocopherol and the oxidized/reduced ratio of CoQ9 and CoQ10 did not change in the mTBI group compared to the control group. Conversely, the oxidized/reduced ratio of CoQ9 and CoQ10 and α-tocopherol levels were reduced in the sTBI group compared to the control. However, the observed reductions were different for CoQ9 and CoQ10. Indeed, the authors observed an increase in the reduced and a decrease in the oxidized form of CoQ9, as well as a reduction in both the reduced and oxidized forms of CoQ10. Therefore, the authors suggested that the sTBI-induced alterations in the levels and redox states of CoQ9 and CoQ10 could explain the mitochondrial impairment found after severe traumatic brain injury.

The alteration in the mitochondrial dynamic can also be involved in neurodegenerative pathology development. Rodríguez-Periñán et al. [24] studied frontotemporal lobar degeneration with the TAR-DNA binding protein of 43kDA (TDP-43) inclusions (FTLD-TDP), a neurodegenerative pathology caused by loss-of-function (LOF) mutations in the GRN gene, which encodes progranulin (PGRN). PGRN is a highly conserved secreted protein that is expressed in multiple cell types in CNS and peripheral tissues. PGRN controls cell growth, survival, repair, and inflammation, and plays a significant role in regulating the function of the lysosomes and microglia. In experiments performed on GRN KD SH-SY5Y neuroblastoma cells, the authors found that PGRN deficiency determined mitochondrial depolarization, enhanced ROS production, and reduced ATP levels. Moreover, they also found that PGRN-deficient cells displayed autophagy dysfunction with increased mitochondrial mass and the accumulation of damaged mitochondria. They concluded that PGRN is involved in the lysosomal degradation of damaged mitochondria. In other experiments conducted on lymphoblasts from FTLD-TDP patients carrying an LOF mutation in the GRN gene, the authors found mitochondrial depolarization and lower ATP content to be associated with increased mitochondrial fission to eliminate dysfunctional mitochondria. Finally, treating PGRN-deficient cells with two phosphorylation inhibitors and the cytosolic accumulation of TDP-43 (IGS-2.7 and IGS-3.27) rescued mitochondrial function. The results illuminated the mechanism contributing to the mitochondrial dysfunction in FTLD-TDP associated with LOF GRN mutations and proposed the utilization of molecules targeting TDP-43 as a therapeutic strategy for restoring mitochondrial function.

The mitochondrial capacity to regulate cellular ROS content may be an essential determinant of lifespan. Gröger et al. [25] evaluated mitochondrial involvement in aging processes using experimental models of unicellular eukaryotes spanning a broad range of the Ascomycota clade. They studied chronological lifespan (CLS), defined as the survival of non-proliferating cells. Mitochondrial metabolism plays a key role in regulating CLS, and the upregulation of mitochondrial respiration has been linked to activating stress responses and promoting lifespan. The authors studied the relationship among the (i) longevity, (ii) mitochondrial activity, and (iii) oxidative stress tolerance of 13 different yeasts (12 from the hemiascomycete/budding yeast subdivision and the archiascomycete *S*. *pombe*); they classified the 13 yeast species into four groups based on the results related to CLS, oxidative stress resistance, and their mitochondrial activity. Group 1 exhibited high mitochondrial activity and resistance to oxidative stress, as well as long CLS (*K*. *lactis*, *S*. *bayanus*, and *L*. *elongisporus*). Group 2 exhibited high mitochondrial activity, low resistance to oxidative stress, and low CLS (*S*. *kluyvei*, *K*. *thermotolerans*, *L*. *waltii*, and *Y*. *lipolytica*). Group 3 exhibited intermediate mitochondrial activity, resistance to oxidative stress, and intermediate CLS (*S*. *mikatae*, *S*. *paradoxus*, and *S*. *pombe*). Group 4 exhibited undetectable mitochondrial activity and either elongated lifespan (*S*. *kudiravzevii* and *S*. *cerevisiae*) or high tolerance to H_2_O_2_ (*S*. *castellii*). Overall, the authors concluded that mitochondrial activity cannot anticipate the length of lifespan and showed a relationship between sustained viability and tolerance to H_2_O_2_ stress during the logarithmic phase of growth in most yeasts—long-lived species are more resistant to H_2_O_2_ stress.

The mitochondrial role in the aging process was also deeply analyzed in an interesting review by Maldonado et al. [26]. In the first part of their manuscript, the authors described the aging processes as sustained by a systemic inflammation to which several mechanisms, identified as the nine hallmarks of the aging process, contributed. These processes are telomere shortening, genomic instability, epigenetic modifications, mitochondrial dysfunction, loss of proteostasis, dysregulated nutrient sensing, stem cell exhaustion, cellular senescence, and altered cellular communication. In the second part of the paper, the authors focused on the mitochondria’s involvement in the aging process at the center of oxidative metabolism (ATP production) and the leading site of ROS production. The authors focused on the molecular mechanisms and pathways activated by mitochondrial ROS and ROS derived from other endogenous and exogenous factors that can affect antioxidant defenses. These events can promote oxidative stress and damage different biomolecules, affecting inter- and intra-cellular signaling and gene expression pathways and causing several cell alterations. They concluded that the interplay between these events could lead to aging, which increases aging-associated syndromes and aging-related diseases. In this context, antioxidant supplementation can slow down the aging process. In the last part of the review, the authors also highlighted the relevance of combining the biomarkers obtained from OMICS studies and machine-learning approaches to obtain data that can potentially be used to construct highly accurate aging clocks to predict biological age. The biomarkers could be used as a target for therapeutic interventions to improve health and extend the human lifespan.

Finally, in an excellent review by Ježek [27], acute redox signaling from mitochondria to the cytosol was discussed. It is known that the balance between pro-oxidant and antioxidant processes determines the redox state within the cell and compartments and/or organelles. When ROS production increases or antioxidant mechanisms decrease, oxidative stress occurs above a certain threshold, which is distinct in different cell types. However, below such oxidative stress thresholds, there are transient increases in ROS, typically increases in H_2_O_2_, which represent redox signals that act within the same single compartment (mitochondria) or between distinct compartments (mitochondria and cytosol and/or plasma membrane) that cause only post-translational modifications in a range of minutes, which does not allow for the reprogramming of the transcriptome. The author focused their attention on the possible role of peroxiredoxin 3 in the signaling from mitochondria to cytosol, and mitochondrial matrix MnSOD-derived H_2_O_2_ that diffuses into the cytosol to take part in the signaling from the mitochondria to cytosol and plasma membrane (redox signaling upon insulin secretion, stimulated either with glucose, ketoisocaproate, or fatty acid).

Overall, the articles published in our Special Issue highlight the central role of mitochondria and their dysfunctions in the development and progression of pathologies affecting different tissues, including organelles involved in metabolic functions and the primary sources of ROS. Specifically, all manuscripts have added new information on the mechanisms involved, highlighting ROS’ central role as signal molecules in signaling pathways that can be significantly altered when a redox imbalance occurs.
